# Pre-stretching of the Hamstrings Before Squatting Acutely Increases Biceps Femoris Thickness Without Impairing Exercise Performance

**DOI:** 10.3389/fphys.2020.00769

**Published:** 2020-07-07

**Authors:** Thiago Barbosa Trindade, Leônidas Oliveira Neto, José Claudino Neto Pita, Vagner Deuel de Oliveira Tavares, Paulo Moreira Silva Dantas, Brad J. Schoenfeld, Jonato Prestes

**Affiliations:** ^1^Graduation Program in Physical Education, Catholic University of Brasilia, Brasilia, Brazil; ^2^Department of Arts, Federal University of Rio Grande do Norte, Natal, Brazil; ^3^Graduation Program in Physical Education, Federal University of Rio Grande do Norte, Natal, Brazil; ^4^Laboratory of Hormone Measurement, Department of Physiology and Behavior, Federal University of Rio Grande do Norte, Natal, Brazil; ^5^Department of Health Sciences, CUNY Lehman College, Bronx, NY, United States

**Keywords:** stretching, muscle strength, electromyography, hamstrings, muscle thickness

## Abstract

**Background:** Bilateral squat exercise is widely used in resistance training (RT) programs to increase lower limb strength and muscle mass, but this exercise does not result in significant hypertrophy of the hamstrings. It has been speculated that stretching between sets with a certain degree of tension results in muscle hypertrophy, while acute stretching could decrease performance during maximal contractions.

**Objective:** This study investigated the acute effects of hamstring stretching before bilateral squatting on muscle thickness (MT), electromyography (EMG), and total training volume (TTV) on exercise performance.

**Methods:** Fourteen resistance-trained young men, with ∼7.5 years of RT experience, performed the 10 repetition maximum (RM) for the barbell squat in two sessions (test–retest) separated by period after 48 h. Participants engaged in two resistance exercise conditions separated by a 1 week recovery interval: one session employed hamstrings stretching and the other did not include hamstrings stretching. Before and after each resistance exercise session, the thickness of the quadriceps muscles and biceps femoris long head were obtained by ultrasound imaging. Moreover, the EMG amplitudes for the quadriceps muscles, biceps femoris, and iliocostalis muscles were recorded during back squat performance. The TTV was also evaluated for each exercise session.

**Results:** A significant increase in MT was observed after every set in both conditions for the evaluated quadriceps muscles (all *p* < 0.05), while for the biceps femoris, this effect was found only in the stretching condition (*p* < 0.05). EMG activity increased in the rectus femoris, vastus lateralis, and vastus medialis for the stretching condition. For the non-stretching condition, activity only increased in the vastus lateralis and medialis. There was no difference in EMG activity for the biceps femoris and iliocostalis in both conditions.

**Conclusion:** Stretching the hamstrings immediately before each set of the back squat can be used to acutely increase biceps femoris thickness without impairing squat performance.

## Introduction

Resistance training (RT) is widely recommended for subjects seeking improvements of the neuromuscular system, athletic performance, and overall health and wellness (American College of Sports Medicine, 2009; Rønnestad and Mujika, 2014; Jewiss et al., 2016; Lesinski et al., 2016; Lopez et al., 2018). Men and women of all ages display increases in muscle hypertrophy, muscle strength, and functional capacity with the performance of regimented RT (Damas et al., 2015; Prestes et al., 2015; Papa et al., 2017).

The practice of RT can improve flexibility (Potier et al., 2009) especially with the inclusion of stretching exercises (Junior et al., 2017); however, there is limited evidence regarding the use of stretching during RT. Several studies have investigated the acute and chronic responses of agonist muscle stretching on strength, flexibility, hypertrophy, total training volume (TTV), and metabolic stress (Souza et al., 2013; Junior et al., 2017; Evangelista et al., 2019; Marin et al., 2019). Miranda et al. (2015) evaluated the effect of antagonist (pectoralis major) muscle stretching, performed during the interset rest period, on seated row performance in resistance trained men. Antagonist muscle stretching resulted in more repetitions in the seated row and higher electromyographic (EMG) activity of the latissimus dorsi and biceps brachii as compared with a non-stretching condition. Sandberg et al. (2012) also reported an increase in torque production of the knee extensors and vertical jump performance following static stretching of the antagonist muscles. Moreover, Simpson et al. (2017) demonstrated an increase in fascicle length, lateral and medial gastrocnemius muscle thickness (MT), and a decrease in penation angle following 6 weeks of moderate-intensity static stretch training. Although some stretching approaches during RT may decrease muscle performance, there is a paucity of research investigating the effects of stretching the antagonist muscles on lower limb performance during squatting in trained subjects.

Squat exercise is widely used in RT programs to increase lower limb strength and muscle mass (Fonseca et al., 2014; Earp et al., 2015). Although the concentric phase of the squat involves hip extension, squatting does not result in significant hypertrophy of the hamstrings (Bloomquist et al., 2013; Kubo et al., 2019), conceivably due to the relative absence of change in the muscle’s length-tension curve during the movement (Sugisaki et al., 2014). It is speculated that interset stretching to a certain level of mechanical tension results in muscle hypertrophy (Nunes et al., 2020), while acute, passive stretching could decrease performance during maximal contractions (Magnusson and Renström, 2006).

Several measures are used to evaluate the effects of an RT session. One such measure is volume load, defined as the product of repetitions and lifted load [number of repetitions × load (kg)], which is considered a useful marker to estimate workload in a training session (Schoenfeld et al., 2018). Another common measure, surface EMG, is a non-invasive tool used to evaluate muscle activation (Gabriel et al., 2006). Finally, B-mode ultrasound is a validated method for measuring MT, which is correlated with muscle cross-sectional area, a hypertrophy parameter (Ema et al., 2013; Timmins et al., 2016), as well as acutely assessing skeletal muscle cell swelling in response to a RT bout (de Almeida et al., 2019). Intriguingly, a recent study by Hirono et al. (2020) found that acute measures of muscle cell swelling were positively correlated with chronic increases in muscle hypertrophy.

There is a dearth of scientific evidence as to the effects of hamstring stretching prior to performance of the bilateral squat, especially in regard to muscle activation and exercise performance. Given the aforementioned evidence that stretching may elicit muscular adaptations, this remains a gap in the literature. Thus, the objective of the present study was to evaluate the acute effects of hamstrings stretching before performance of the back squat on MT, EMG, and TTV. We hypothesized that the hamstrings stretching performed before sets of the back squat would reduce the EMG, and increase MT of the stretched muscles; and would increase agonist muscles recruitment; accompanied by a higher total training.

## Materials and Methods

### Study Design

**FIGURE 1:**
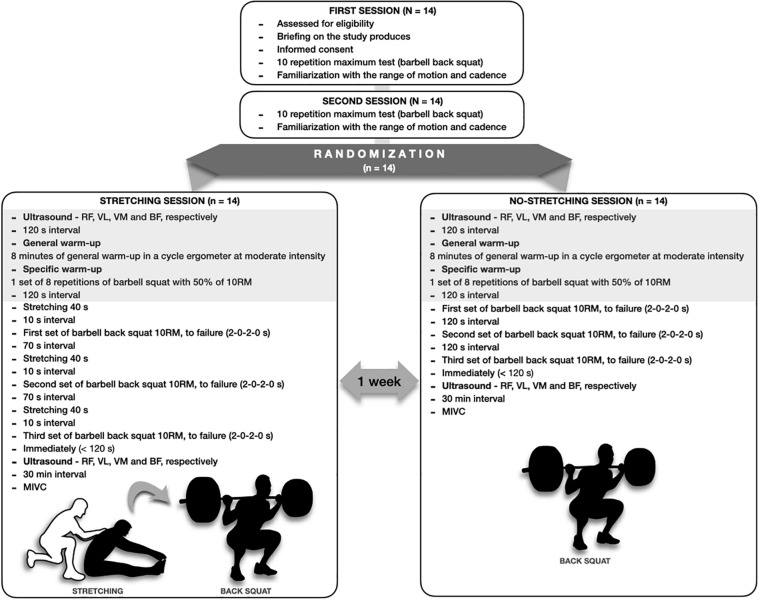
Experimental design.

In an effort to evaluate the effects of hamstrings stretching on muscle performance in an RT session, subjects with previous experience in squat exercise were submitted to two acute training sessions in a randomized order: (1) with stretching before sets and (2) without stretching. All subjects were familiar with the technique and cadence used in the training sessions, which consisted of three sets of the barbell back squat performed to momentary muscle failure. Before and after each experimental session, the thicknesses of the quadriceps muscles (except for the vastus intermedius), and biceps femoris were obtained by ultrasound imaging. During squatting, the activity of the rectus femoris, vastus lateralis, vastus medialis, biceps femoris, and iliocostalis muscles was assessed by EMG. The TTV for each session was also assessed. The load for 10 repetition maximum (RM) was obtained in two separate sessions with a minimum recovery interval of 48 h (see Figure 1).

### Participants

Fourteen men (see Table 1) with ∼7.5 years of RT experience participated in this study. All subjects were habituated to perform the barbell back squat exercise, and had no history of osteomyoarticular injuries or limitations that could impair exercise performance. The study was approved by the local Research Ethics Committee (No.: 2,110,224), and followed the guidelines for data collection in human beings established in the resolution No. 466/12, of 12/12/12 by the National Health Council. All subjects were informed about the procedures, risks, and benefits of the investigation, and gave their signed informed consent prior to participation.

**TABLE 1:** Subjects’ characteristics.

Variables	Mean (CI) (*n* = 14)
Age (years)	28.46(25−31)
Weight (kg)	80.22(74−85)
Height (cm)	174(169−179)
Body mass index	26.34(25−27)
10RM Squat (kg)	103(91−116)
Relative 10RM Squat	1.29(1.1−1.4)
Training experience (years)	7.53(3.6−11)

*10RM, 10 repetition maximum.*

### 10 Repetition Maximum (10RM) Testing

The 10RM test was performed in the squat exercise to determine the exact training load for each individual subject. The subjects previously performed a familiarization session in order to reproduce the exercise technique under the supervision of three physical education professionals. For the test, subjects performed a warm-up consisting of 8 min of stationary cycle ergometry at a light intensity, followed by a set of eight repetitions with 60% of the load equivalent to 10RM (estimated). Each subject initiated the test with the suggested load for 10RM. When the subject reached the goal of 10 repetitions, the test was interrupted, and the load increased in 5–10% increments until ascertaining the 10RM. The 10RM was determined within three attempts with a minimum interval of 5 min between attempts. A metronome was used to control the velocity of the movement, establishing a cadence of 2 s for the concentric phase, and 2 s for the eccentric phase, without interruption in the transitions between them. The amplitude of the movement was limited to a depth in which the subject’s femur was parallel to the ground, demarcated with the aid of a tape. During the 10RM test attempts, verbal encouragement was provided to encourage volunteers to employ maximum effort. To ensure test reliability, the same procedure was repeated after 48 hours, with a high intraclass correlation coefficient (ICC) found between the first and second tests (*r* = 0.981; *p* < 0.0001).

### Training Sessions

For the experimental sessions, all subjects completed two resistance exercise conditions in a randomized order separated by a 7-day recovery period. Both resistance exercise conditions (stretching and non-stretching) consisted of 8 min of a general warm-up using a cycle ergometer at moderate intensity followed by one set of eight repetitions of the barbell squat with 50% of the subject’s 10RM. Subjects then proceeded to complete three sets of the barbell back squat at their 10RM, maintaining a cadence of 2 s for the concentric phase and 2 s for the eccentric, with an interset rest interval of 120 s.

### Stretching Session

Subjects completed a static stretch for the hamstrings just before performance of the barbell squat. A research volunteer manually provided support to the participant’s shoulder blades, exerting gradual scapular pressure. All participants were required to report a perception of pain higher than 7 (range from 0 to 10) during the active rest with static stretching. The static stretching position was sustained for 40 s, in a sitting position, with the knees extended and hips flexed. Within 10 s after completion of the stretching protocol, participants performed the barbell squat (10RM to failure), then rested for 70 s and repeated the process three times. The total recovery time was 120 s, consisting of 70 s of passive rest, 40 s active rest, and 10 s of transition to performance of the barbell squat exercise.

### Non-stretching Session

For the non-stretching session, the participant completed the barbell squat (10RM to failure) followed by a passive recovery of 120 s for all three sets.

### Muscle Thickness Assessment

**FIGURE 2:**
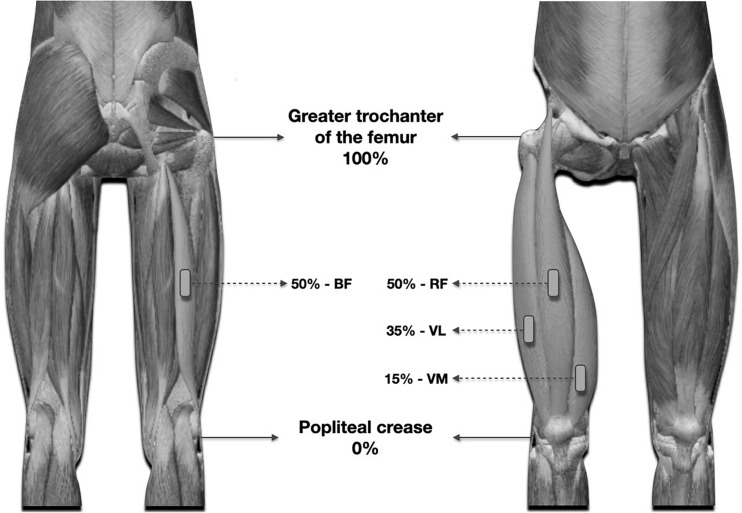
Illustration of the anatomical points used for ultrasound measurements and fixation of surface electrodes, for the evaluation of EMG.

**FIGURE 3:**
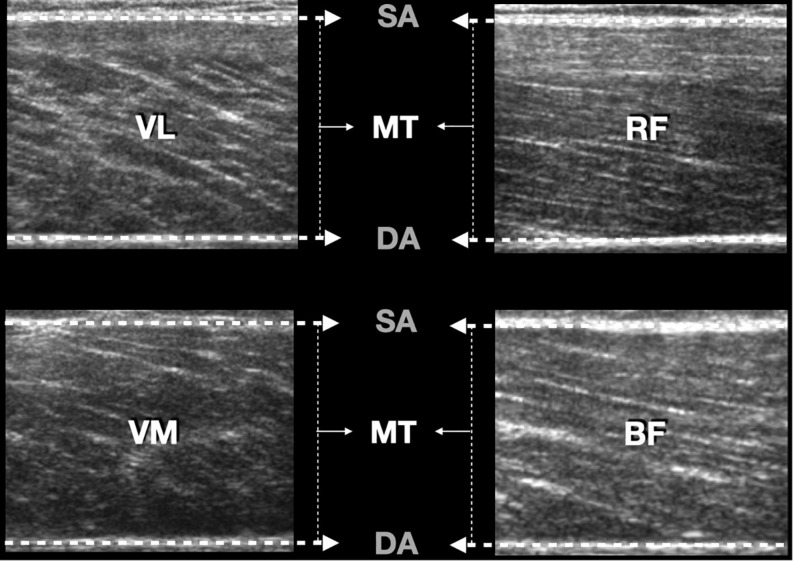
Example of ultrasound image showing the cross-cutting scans of the muscles. SA, superficial aponeurosis; DA, deep aponeurosis; MT, muscle thickness; VL, vastus lateralis; RF, rectus femoris; VM, vastus medialis; BF, biceps femoris.

Before and after each training session, the thickness of the quadriceps femoris muscles (except for the vastus intermedius muscle), and biceps femoris were evaluated by the same technician by images obtained using B-mode ultrasonography (Medison SA-9900, Live 4D; Samsung Medison Co., Ltd.; Gyeonggi-Do, South Korea) with a probe of 100 mm, 10–15 MHz. Ultrasound images before RT sessions were recorded at a specific joint angle (150°), which corresponds to the approximate total excursion of the knee extension (180°) with the subject in the supine position. Before each scanning session, the subject remained at rest for 20 min. For each site of interest (see Figure 2), the transducer was initially positioned transversely to visualize the greater distance between the internal border of the superior and inferior aponeuroses. The transducer then was aligned in the fascicular plane to visualize the muscle of interest on the ultrasound monitor. To facilitate the identification of the anatomical sites analyzed in each of the evaluated muscles, as well as enhancing reproducibility of the tests (pre and post intervention), the analyzed regions were demarcated with henna ink. MT was measured as the distance between the superficial aponeurosis and the deep aponeurosis (see Figure 3). To ensure accuracy of the measurements, at least three images were obtained from each evaluated site. For each measure of MT, the image was frozen and a preliminary measurement was made in the ultrasound unit. This procedure was repeated two more times and, if a difference superior to 1 mm between measures was found, a fourth image was obtained. After this, the measures were saved on hard drive for posterior analysis in ImageJ 1.42 q software (National Institutes of Mental Health, United States). After obtaining measurements of the quadriceps muscles, the subject was positioned in the prone position to measure the thickness of the biceps femoris muscle. The measures followed this order: RF, VL, VM, and BF. The post-session evaluations were performed approximately 120 s after the last squat set, which equated to the time required for the participant to get positioned on the stretcher (located in the same environment in which the exercises were performed). The total time to obtain all measurements was approximately 20 min. All subjects were instructed to refrain from drinking alcoholic beverages, and practicing physical exercises in the 72 h preceding the exams.

The measurement regions were determined on the basis of the following parameters:

• Vastus lateralis: 35% of the thigh length; popliteal fold (0%) to the major trochanter (100%);
• Vastus medialis: 15% of thigh length;
• Rectus femoris: 50% of the thigh length;
• Biceps femoris: 50% of the thigh length; popliteal fold (0%) to the major trochanter (100%).

Muscle thickness measurements were quantified with the aid of image analysis software ImageJ 1.42 q (National Institutes of Mental Health, United States). Three images were obtained for each site, and the intra-class correlation value was calculated for each measure based on the pre-exercise measures.

### Electromyographic Activity

The measures of EMG activity were evaluated by surface electrodes with an interelectrode spacing of 2 cm fixed on the right side of the body at the same points used for the assessment of MT: vastus lateralis, vastus medialis, rectus femoris, and biceps femoris. EMG activity was also recorded for the iliocostalis muscle, with the electrodes attached three centimeters lateral to the L3 spinous process and the reference electrode positioned on the olecranon process of ulna. The same evaluator fixed pairs of surface electrodes Ag/AGCL (model 2223BRQ, 3M brand), on the bellies of the analyzed muscles, parallel to the striations of the respective muscle fibers, after cleaning and trichotomization of the skin surface.

The maximal voluntary isometric contraction (MVIC) was used as a reference to normalize EMG signals. First, the subjects were instructed to perform maximum voluntary isometric contractions for all analyzed muscles. For the vastus lateralis, vastus medialis, and rectus femoris muscles, subjects remained with knee extension limited to 60°; for the biceps femoris, they maintained a knee flexion of 60°, and for the iliocostalis, they maintained hyperextension of the trunk. The MVIC of the quadriceps, biceps femoris, and iliocostalis muscles were obtained by manual resistance in an extensor chair, a flexor table, and a 45° hip extension machine, respectively, all of the same brand (Physicus^®^, São Paulo, Brazil). Furthermore, straps were used to limit the aforementioned angles of flexion and extension, with the exception of trunk extension, for which manual resistance was employed. Finally, two attempts of MVIC with 5 s of duration were performed at each position. The highest RMS-EMG value obtained from the two tests for MVIC was used for EMG normalization.

The described positions and angles were obtained with the support of an extensor chair, a unilateral knee flexor, and a back extension free machine, respectively. The RMS-EMG values during performance of three sets of the barbell squat to momentary muscle failure were recorded and the mean of the values was normalized by RMS-EMG during MVIC as %MVIC. EMG activity was evaluated with the aid of Myotool [Miotec^®^ eight-channel EMG, Porto Alegre/RS, Brazil], with a sampling frequency of 1000 Hz, 2000-fold gain and bipolar surface electrodes. Data analysis was performed using the Miograph 2.0 software, with a notch filter of 60 Hz, high pass of 20 Hz, and low pass of 500 Hz.

### Total Training Volume

Total training volume was calculated using the following equation: TTV = number of sets × number of repetitions × weight. The TTV was assessed for each session to compare the performance obtained by the subject during both training sessions.

### Data Analysis

The Shapiro–Wilk test was used to verify data normality and Levene’s test was performed to check data homogeneity. All data are presented as means (M) and confidence intervals (CI). We applied an ICC, models (2,2) with values (<0.49 poor, 0.50–0.74 moderate, 0.75–0.89 good, and >0.90 excellent) (Trevethan, 2017; Liljequist et al., 2019; Portney, 2020) for the measurement of pre-exercise MT and 10RM tests for both conditions. TTV was compared between training sessions by an independent *t*-test. Between-session values of MT (two groups × two moments) and EMG (two groups × three moments) amplitude were compared by the use of a two-way ANOVA. Significant main effects and interactions were further analyzed with the Bonferroni’s *post hoc* test. Whenever the sphericity assumption was violated, the Greenhouse-Geisser correction was employed. The partial eta squared (η^2^*_p_*) was used as a measure of effect size and to represent the proportion of variance in the data that is attributed to the independent variable. Statistical analyses were carried out using SPSS v.23.0 (SPSS, Inc., Chicago, IL, United States). A *p < 0.05* was considered statistically significant.

## Results

### Total Training Volume

There was no difference in TTV between conditions stretching vs non-stretching [*F* = 0.260; (*t*) = 5, (*df* = 26); *p* < 0.611]. TTV stretching = 2172 kg (1848–2495); TTV non-stretching = 2278 kg (1973–2582).

### Intraclass Correlation Coefficient

The ICCs for pre-exercise MT were: rectus femoris (*r* = 0.998; *p* < 0.0001); vastus lateralis (*r* = 0.999; *p* < 0.0001); vastus medialis (*r* = 0.998; *p* < 0.0001); femoris biceps (*r* = 0.996 *p* < 0.0001).

### Muscle Thickness

There was an effect of the exercise session in increasing MT of the rectus femoris [*F*_(1;26)_ = 27.51; *p* < 0.0001; η^2^*_p_* = 0.516], vastus lateralis [*F*_(1;26)_ = 40.97; *p* < 0.0001; η^2^*_p_* = 0.612], and vastus medialis [*F*_(1;26)_ = 177.3; *p* < 0.0001; η^2^*_p_* = 0.872] for both groups. There was an effect of sets post exercise for the biceps femoris in the stretching condition [*F*_(1;26)_ = 31.13; *p* < 0.0001; η^2^*_p_* = 0.545]. Bonferroni’s *post hoc* showed that the MT increased after every session in both conditions (all *p*-values < 0.05), except for the biceps femoris in the non-stretching condition (*p* > 0.05). No interaction for time was observed between conditions for the rectus femoris [*F*_(1:10)_ = 0.169; *p* < 0.689; η^2^*_p_* = 0.017], vastus lateralis [*F*_(1;10)_ = 3.36; *p* < 0.096; η^2^*_p_* = 0.252], vastus medialis [*F*_(1;10)_ = 5.31; *p* < 0.054; η^2^*_p_* = 0.347], but a difference was observed for the biceps femoris between post-stretching across time to non-stretching pre and post [*F*_(1;10)_ = 30.54; *p* < 0.0001; η^2^*_p_* = 0.753] (see Table 2).

**TABLE 2:** Muscle thickness analysis.

	Stretching session (*n* = 14)		Non-stretching session (*n* = 14)	
	Mean (CI)	Mean (CI)	Mean (CI)	Mean (CI)
		
Muscle	Pre	Post	Pre	Post
RF (cm)	2.74 (2.5–2.9)	2.80 (2.6–3.0)*	2.73 (2.5–2.9)	2.80 (2.6–3.0)*
VL (cm)	2.95 (2.7–3.1)	3.08 (2.8–3.3)*	2.96 (2.7–3.1)	3.09 (2.8–3.3)*
VM (cm)	3.46 (3.1–3.7)	3.90 (3.6–4.2)*	3.47 (3.2–3.8)	3.79 (3.5–4.1)*
BF (cm)	2.70 (2.4–2.9)	2.84 (2.6–3.0)*^,^**	2.69 (2.4–2.8)	2.72 (2.4–2.8)

CI, confidence interval; RF, rectus femoris; VL, vastus lateralis; VM, vastus medialis; BF, biceps femoris; *difference across time; **difference across condition.

### Electromyography Activation

An effect of sets was seen in EMG for both conditions (pre vs post) on vastus lateralis [*F*_(2;52)_ = 12.42; *p* < 0.0001; η^2^*_p_* = 0.323], rectus femoris [*F*_(2;52)_ = 3.69; *p* < 0.032; η^2^*_p_* = 0.124], vastus medialis [*F*_(2;52)_ = 26.25; *p* < 0.0001; η^2^*_p_* = 0.502]. Bonferroni’s *post hoc* showed that EMG increased RF in set 2 and set 3 compared with set 1 in the stretching condition (all *p*s < 0.05). Moreover, EMG activity increased for the vastus lateralis in set 3 compared with set 1 in both conditions (*p* < 0.05). Also, EMG activity increased for the vastus medialis in set 3 compared with set 2 and set 1 in the stretching condition and in set 3 and set 2 compared with set 1 in the non-stretching condition (see Table 3). No group or time interaction was observed for the biceps femoris [*F*_(2;52)_ = 5.29; *p* < 0.059; η^2^*_p_* = 0.169] and iliocostalis in any condition [*F*_(2;52)_ = 0.876; *p* < 0.422; η^2^*_p_* = 0.033]. No interaction was observed for conditions vs time for the vastus lateralis [*F*_(5;65)_ = 0.736; *p* < 0.599; η^2^*_p_* = 0.054], rectus femoris [*F*_(5;65)_ = 1.44; *p* < 0.100; η^2^*_p_* = 0.221], vastus medialis [*F*_(5;65)_ = 2.74; *p* < 0.105; η^2^*_p_* = 0.174], biceps femoris [*F*_(5;65)_ = 0.941; *p* < 0.461; η^2^*_p_* = 0.067], and iliocostalis [*F*_(5;65)_ = 2.13; *p* < 0.072; η^2^*_p_* = 0.141].

**TABLE 3:** Electromyographic analysis adjusted for the percent of maximum voluntary isometric contraction (%MVIC).

	Stretching session (*n* = 14)					Non-stretching session (*n* = 14)				
			Mean (CI)					Mean (CI)		
		
Muscle	RF	VL	VM	BF	IC	RF	VL	VM	BF	IC
Set 1	47.9 (33–62)	58.6 (45–71)	62.0 (52–71)	21.3 (16–26)	65.5 (50–80)	48.2 (34–61)	58.9 (45–71)	59.2 (49–68)	22.2 (17–27)	61.4 (46–76)
Set 2	52.6 (36–68)^A^	62.6 (48–76)	66.3 (56–76)^A^	21.9 (16–26)	68.9 (54–83)	49.5 (35–63)	60.5 (46–74)	63.2 (53–73)^A^	23.7 (18–28)	61.1 (46–76)
Set 3	52.1 (38–65)^B^	66.0 (53–79)^B^	71.1 (59–82)^B,C^	22.6 (17–27)	70.6 (55–85)	49.4 (37–61)	64.0 (51–77)^B^	66.3 (55–77)^B^	24.2 (19–29)	61.9 (47–76)

*Note: RF, rectus femoris; VL, vastus lateralis; VM, vastus medialis; BF, biceps femoris; IC, iliocostalis; CI, confidence interval; Different letters mean significant difference between sets within conditions (*p* < 0.05). ^*A*^Set 2 difference for Set 1; ^*B*^Set 3 for set 1; ^*C*^Set 3 for Set 2.*

## Discussion

This study assessed the influence of static stretching immediately before the squat exercise on TTV, electromyographic activity, and thickness of primary and synergistic skeletal muscles. The main results from the present study partially confirmed our initial hypothesis in that hamstrings stretching before sets of the barbell back squat resulted in an increased biceps femoris thickness, not observed in the condition without stretching. Squatting with or without stretching increased vastus lateralis, vastus medialis, and rectus femoris thickness, without differences in TTV. All muscles displayed an increase in EMG, regardless of whether stretching was employed. These results highlight the possibility that stretching the hamstrings prior to the bilateral squat does not impair performance and may promote a positive stimulus to the hamstring muscles. This strategy is both time-efficient and easily applied during resistance exercise sessions.

The acute increase in biceps femoris thickness in the stretching condition may be attributed to an increased hyperemia resultant to stretch-induced blood flow restriction and consequent metabolite accumulation, factors that promote muscle cell swelling (e.g., “the pump”) (Schoenfeld, 2013b). This phenomenon might be contributing stimuli to muscle growth (Hirono et al., 2020). Moreover, an association exists between the acute “muscle pump” and integrin activation; a membrane protein that can trigger muscle protein synthesis and thus enhance hypertrophic adaptations (Wackerhage et al., 2019). However, despite the correlation between acute and chronic changes in MT (Wakahara et al., 2012, 2013), it remains to be determined if the increase in biceps femoris thickness found in the present study would result in a chronic increase of muscle mass. In support of the hypothesis, 6 weeks of a static stretching of the triceps surae increased 5.6% the lateral and medial gastrocnemius thickness in detrained subjects (Simpson et al., 2017).

The RT session of the barbell squat without stretching did not increase MT of the biceps femoris. This was expected due to the limited contribution of the hamstrings during bilateral squatting (Ebben, 2009; Bloomquist et al., 2013; Kubo et al., 2019). Muscle length of the biceps femoris remains relatively unaltered during this movement given its dual role in extending the hip and flexing the knee, and thus is not sufficiently stimulated to promote a significant increase in MT (Sugisaki et al., 2014).

The MT of all quadriceps muscles increased in response to squat training, regardless of condition, probably as a result of the muscle pump mechanisms mentioned earlier (Schoenfeld, 2013b; Goto et al., 2019). The acute increase in RF thickness in both resistance exercise conditions may be attributed to a swelling effect after performance of the bilateral squat. Although the length-tension curve of the RF is not significantly modified during the bilateral squat, this should not be confused as an absence of recruitment, as opposed to the higher activation of the vastus muscles (Sugisaki et al., 2014). Importantly, attempting to extrapolate acute measures to chronic adaptations should be done cautiously as only modest chronic hypertrophic responses of the RF are observed in the back squat regardless of the intensity, speed, and range of motion utilized (Fonseca et al., 2014; Earp et al., 2015; Kubo et al., 2019).

Stretching induces alterations in the recruitment pattern of primary muscles and antagonists (Miranda et al., 2015). Thus, stretching the hamstrings prior to performing the back squat may interfere with quadriceps muscle behavior (Sandberg et al., 2012). Our data revealed that despite the absence of differences between conditions (stretching and non-stretching), EMG amplitude of the rectus femoris increased only in the stretching session. We observed increased EMG activity of the rectus femoris in the second and third sets of barbell squat performed after the stretching of the hamstring. Conceivably, this can be attributed to the synergistic relationship of this muscle with the elongated hip extensors. During squatting, the rectus femoris acts as a transmission arm of the force generated by the hip extensors, especially the gluteus maximus, to the knee joint (Kapandji, 1999). Therefore, as the hip extensors are elongated, the rectus femoris begins to withstand greater demands to ensure the maintenance of exercise performance during the supposed reduction in the action of synergic-antagonist hip extensors. A proposed mechanism for this phenomenon is related to mechanical adaptations, such as a gradual decrease in torque production by the hip extensor muscles, as they were stretched before the sets of squat; passively due to viscoelastic stress relaxation, passive torque, and muscle stiffness reduction (Sharman et al., 2006). Alternatively, the torque might actively decrease as consequence of decreased recruitment or reflex sensitivity (Ogura et al., 2007) this seems less likely for the biceps femoris, as EMG activity was not decreased even in the stretching condition. This hypothesis remains speculative.

Pre-exercise stretching of the hamstrings did not alter TTV during squatting. Both Paz et al. (2012) and Miranda et al. (2015) reported an increase in total repetitions completed in the seated row when the pectoralis muscle was stretched between sets. Among the explanatory mechanisms, the authors hypothesized that stretching of the antagonists could decrease coactivation of these muscles, increase the storage of elastic energy in the agonists, and promote alterations in the acute sensitivity of specific muscle proprioceptors (Golgi tendon organs and muscle spindles), thereby increasing performance. However, this would not seem to apply to our findings, as the hamstrings can be regarded as synergists during the bilateral squat exercise (Ebben, 2009). Thus, the stretching did not interfere with biceps femoris EMG during squatting, which may have resulted in the absence of changes in iliocostalis activation between conditions (see Table 2). Thus, it can be inferred that the acute increase in MT observed in the biceps femoris is not directly associated with the magnitude of EMG amplitude during the stretching session (Sugisaki et al., 2014). The EMG represents the neural drive to the muscle (Gabriel et al., 2006) while MT may be associated with metabolite accumulation produced during the stress imposed by the exercise (Schoenfeld, 2013a).

The present study is not without limitations. First, the acute nature of the thickness measures may not reflect chronic adaptations. Second, measures of MT were taken from a specific muscle site, and it is thus unclear as to whether differential results may have occurred along the length of the muscle belly. Third, although MT measures (post-exercise) were taken immediately after the last set of squat (<120 s), following the same order (RF, VL, VM, and BF), it is necessary to recognize that the time between the end of the sessions and the measures might interfere in determining acute MT values (Hirono et al., 2020). Finally, the stretching technique chosen for the present study may not be the most efficient for targeting the hamstrings. Notably, RF, VL, and VM muscles originate from the sciatic tuberosity, and the increased tension during stretching can generate a posterior inclination of the pelvis, which in turn can reduce the intensity of stretching. Moreover, individuals with severe hamstring shortening may increase flexion in the thoracic spine as a compensatory strategy to facilitate performance (López-Miñarro and Rodríguez-García, 2010). Other stretching techniques potentially could produce disparate findings. Future studies should endeavor to investigate EMG and MT in the gluteus maximus and other hamstring muscles, in conjunction with different stretching strategies.

## Conclusion

This study suggests that the stretching of the hamstrings immediately before each set of the bilateral squat can be used to optimize time during a resistance exercise session with the inclusion of flexibility exercises, without impairment of exercise performance. Moreover, the stretching of the hamstrings immediately before each set of the squat may be used to acutely increase biceps femoris thickness. It remains to be determined if the acute stretching-induced increase in biceps femoris thickness enhances muscle hypertrophy when performed chronically over time.

## Data Availability Statement

The raw data supporting the conclusions of this article will be made available by the authors, without undue reservation.

## Ethics Statement

The studies involving human participants were reviewed and approved by the Research Ethics Committee of Federal University of Rio Grande do Norte, Brazil. The patients/participants provided their written informed consent to participate in this study.

## Author Contributions

All authors contributed significantly to this study and read and approved the submitted manuscript.

## Conflict of Interest

The authors declare that the research was conducted in the absence of any commercial or financial relationships that could be construed as a potential conflict of interest.

## References

[B1] American College of Sports Medicine (2009). Progression models in resistance training for healthy adults. *Med. Sci. Sports Exerc.* 41 687–708. 10.1249/MSS.0b013e3181915670 19204579

[B2] Bloomquist K. Langberg H. Karlsen S. Madsgaard S. Boesen M. Raastad T. (2013). Effect of range of motion in heavy load squatting on muscle and tendon adaptations. *Eur. J. Appl. Physiol.* 113 2133–2142. 10.1007/s00421-013-2642-7 23604798

[B3] Damas F. Phillips S. Vechin F. C. Ugrinowitsch C. (2015). A review of resistance training-induced changes in skeletal muscle protein synthesis and their contribution to hypertrophy. *Sport. Med.* 45 801–807. 10.1007/s40279-015-0320-0 25739559

[B4] de Almeida F. N. Lopes C. R. Conceição R. M. da Oenning L. Crisp A. H. de Sousa N. M. F. (2019). Acute effects of the new method sarcoplasma stimulating training versus traditional resistance training on total training volume, lactate and muscle thickness. *Front. Physiol.* 10:579. 10.3389/fphys.2019.00579 31156459 PMC6529514

[B5] Earp J. E. Newton R. U. Cormie P. Blazevich A. J. (2015). Inhomogeneous quadriceps femoris hypertrophy in response to strength and power training. *Med. Sci. Sports Exerc.* 47 2389–2397. 10.1249/MSS.0000000000000669 25811947

[B6] Ebben W. P. (2009). Hamstring activation during lower body resistance training exercises. *Int. J. Sports Physiol. Perform.* 4 84–96. 10.1123/ijspp.4.1.84 19417230

[B7] Ema R. Wakahara T. Miyamoto N. Kanehisa H. Kawakami Y. (2013). Inhomogeneous architectural changes of the quadriceps femoris induced by resistance training. *Eur. J. Appl. Physiol.* 113 2691–2703. 10.1007/s00421-013-2700-1 23949789

[B8] Evangelista A. L. De Souza E. O. Moreira D. C. B. Alonso A. C. Teixeira C. V. L. S. Wadhi T. (2019). Interset stretching vs. traditional strength training: effects on muscle strength and size in untrained individuals. *J. strength Cond. Res.* 33(Suppl. 1), S159–S166. 10.1519/JSC.0000000000003036 30688865

[B9] Fonseca R. M. Roschel H. Tricoli V. De Souza E. O. Wilson J. M. Laurentino G. C. (2014). Changes in exercises are more effective than in loading schemes to improve muscle strength. *J. Strength Cond. Res.* 28 3085–3092. 10.1519/JSC.0000000000000539 24832974

[B10] Gabriel D. A. Kamen G. Frost G. (2006). Neural adaptations to resistive exercise. *Sport. Med.* 36 133–149. 10.2165/00007256-200636020-00004 16464122

[B11] Goto M. Maeda C. Hirayama T. Terada S. Nirengi S. Kurosawa Y. (2019). Partial range of motion exercise is effective for facilitating muscle hypertrophy and function through sustained intramuscular hypoxia in young trained men. *J. Strength Cond. Res.* 33 1286–1294. 10.1519/JSC.0000000000002051 31034463

[B12] Hirono T. Ikezoe T. Taniguchi M. Tanaka H. Saeki J. Yagi M. (2020). Relationship between muscle swelling and hypertrophy induced by resistance training. *J. Strength Cond. Res.* [Online ahead of print]. 10.1519/JSC.0000000000003478 31904714

[B13] Jewiss D. Ostman C. Smart N. A. (2016). The effect of resistance training on clinical outcomes in heart failure: a systematic review and meta-analysis. *Int. J. Cardiol.* 221 674–681. 10.1016/j.ijcard.2016.07.046 27423089

[B14] Junior R. M. Berton R. de Souza T. M. F. Chacon-Mikahil M. P. T. Cavaglieri C. R. (2017). Effect of the flexibility training performed immediately before resistance training on muscle hypertrophy, maximum strength and flexibility. *Eur. J. Appl. Physiol.* 117 767–774. 10.1007/s00421-016-3527-3 28251401

[B15] Kapandji A. I. (1999). *Physiologie Articulaire: Membre Inférieur.* Paris: Maloine.

[B16] Kubo K. Ikebukuro T. Yata H. (2019). Effects of squat training with different depths on lower limb muscle volumes. *Eur. J. Appl. Physiol.* 119 1933–1942. 10.1007/s00421-019-04181-y 31230110

[B17] Lesinski M. Prieske O. Granacher U. (2016). Effects and dose-response relationships of resistance training on physical performance in youth athletes: a systematic review and meta-analysis. *Br. J. Sports Med.* 50 781–795. 10.1136/bjsports-2015-095497 26851290 PMC4941165

[B18] Liljequist D. Elfving B. Roaldsen K. S. (2019). Intraclass correlation – A discussion and demonstration of basic features. *PLoS One* 14:e0219854. 10.1371/journal.pone.0219854 31329615 PMC6645485

[B19] Lopez P. Pinto R. S. Radaelli R. Rech A. Grazioli R. Izquierdo M. (2018). Benefits of resistance training in physically frail elderly: a systematic review. *Aging Clin. Exp. Res.* 30 889–899. 10.1007/s40520-017-0863-z 29188577

[B20] López-Miñarro P. A. Rodríguez-García P. L. (2010). Hamstring muscle extensibility influences the criterion-related validity of sit-and-reach and toe-touch tests. *J. Strength Cond. Res.* 24 1013–1018. 10.1519/JSC.0b013e3181c7c60d 20300025

[B21] Magnusson P. Renström P. (2006). The European College of Sports Sciences position statement: the role of stretching exercises in sports. *Eur. J. Sport Sci.* 6 87–91. 10.1080/17461390600617865

[B22] Marin D. P. Urtado C. B. Marques C. G. Serafim A. I. S. Polito L. F. T. De Almeida F. N. (2019). Effects of inter-set stretching on acute hormonal and metabolic response: a pilot study. *Hum. Mov.* 20 55–61. 10.5114/hm.2019.79218

[B23] Miranda H. Maia M. D. F. Paz G. A. Costa P. B. (2015). Acute effects of antagonist static stretching in the inter-set rest period on repetition performance and muscle activation. *Res. Sport. Med.* 23 37–50. 10.1080/15438627.2014.975812 25630245

[B24] Nunes J. P. Schoenfeld B. J. Nakamura M. Ribeiro A. S. Cunha P. M. Cyrino E. S. (2020). Does stretch training induce muscle hypertrophy in humans? A review of the literature. *Clin. Physiol. Funct. Imaging* 40 148–156. 10.1111/cpf.12622 31984621

[B25] Ogura Y. Miyahara Y. Naito H. Katamoto S. Aoki J. (2007). Duration of static stretching influences muscle force production in hamstring muscles. *J. Strength Cond. Res.* 21 788–792. 10.1519/R-18785.1 17685679

[B26] Papa E. V. Dong X. Hassan M. (2017). Resistance training for activity limitations in older adults with skeletal muscle function deficits: a systematic review. *Clin. Interv. Aging* 12 955–961. 10.2147/CIA.S104674 28670114 PMC5479297

[B27] Paz G. A. de Freitas Maia M. Lima V. P. Oliveira C. G. Bezerra E. Simão R. (2012). Maximal exercise performance and electromyography responses after antagonist neuromuscular proprioceptive facilitation: a pilot study. *J. Exerc. Physiol.* 15 60–67.

[B28] Portney L. G. (2020). *Foundations of Clinical Research: Applications to Evidence-Based Practice.* Philadelphia: FA Davis.

[B29] Potier T. G. Alexander C. M. Seynnes O. R. (2009). Effects of eccentric strength training on biceps femoris muscle architecture and knee joint range of movement. *Eur. J. Appl. Physiol.* 105 939–944. 10.1007/s00421-008-0980-7 19271232

[B30] Prestes J. da Cunha Nascimento D. Tibana R. A. Teixeira T. G. Vieira D. C. L. Tajra V. (2015). Understanding the individual responsiveness to resistance training periodization. *Age* 37:9793. 10.1007/s11357-015-9793-x 25971877 PMC4430497

[B31] Rønnestad B. R. Mujika I. (2014). Optimizing strength training for running and cycling endurance performance: a review. *Scand. J. Med. Sci. Sport* 24 603–612. 10.1111/sms.12104 23914932

[B32] Sandberg J. B. Wagner D. R. Willardson J. M. Smith G. A. (2012). Acute effects of antagonist stretching on jump height, torque, and electromyography of agonist musculature. *J. Strength Cond. Res.* 26 1249–1256. 10.1519/JSC.0b013e31824f2399 22344065

[B33] Schoenfeld B. J. (2013a). Is there a minimum intensity threshold for resistance training-induced hypertrophic adaptations? *Sport. Med.* 43 1279–1288. 10.1007/s40279-013-0088-z 23955603

[B34] Schoenfeld B. J. (2013b). Potential mechanisms for a role of metabolic stress in hypertrophic adaptations to resistance training. *Sport Med.* 43 179–194. 10.1007/s40279-013-0017-1 23338987

[B35] Schoenfeld B. J. Contreras B. Krieger J. Grgic J. Delcastillo K. Belliard R. (2018). Resistance training volume enhances muscle hypertrophy but not strength in trained men. *Med. Sci. Sports Exerc.* 51 94–103. 10.1249/MSS.0000000000001764 30153194 PMC6303131

[B36] Sharman M. J. Cresswell A. G. Riek S. (2006). Proprioceptive neuromuscular facilitation stretching: mechanisms and clinical implications. *Sport Med.* 36 929–939. 10.2165/00007256-200636110-00002 17052131

[B37] Simpson C. L. Kim B. D. H. Bourcet M. R. Jones G. R. Jakobi J. M. (2017). Stretch training induces unequal adaptation in muscle fascicles and thickness in medial and lateral gastrocnemii. *Scand. J. Med. Sci. Sport* 27 1597–1604. 10.1111/sms.12822 28138986

[B38] Souza A. C. Bentes C. M. De Salles B. F. Reis V. M. Alves J. V. Miranda H. (2013). Influence of inter-set stretching on strength, flexibility and hormonal adaptations. *J. Hum. Kinet.* 36 127–135. 10.2478/hukin-2013-0013 23717362 PMC3661884

[B39] Sugisaki N. Kurokawa S. Okada J. Kanehisa H. (2014). Difference in the recruitment of hip and knee muscles between back squat and plyometric squat jump. *PLoS One* 9:e101203. 10.1371/journal.pone.0101203 24979707 PMC4076339

[B40] Timmins R. G. Shield A. J. Williams M. D. Lorenzen C. Opar D. A. (2016). Architectural adaptations of muscle to training and injury: a narrative review outlining the contributions by fascicle length, pennation angle and muscle thickness. *Br. J. Sports Med.* 50 1467–1472. 10.1136/bjsports-2015-094881 26817705

[B41] Trevethan R. (2017). Intraclass correlation coefficients: clearing the air, extending some cautions, and making some requests. *Heal. Serv. Outcomes Res. Methodol.* 17 127–143. 10.1007/s10742-016-0156-6

[B42] Wackerhage H. Schoenfeld B. J. Hamilton D. L. Lehti M. Hulmi J. J. (2019). Stimuli and sensors that initiate skeletal muscle hypertrophy following resistance exercise. *J. Appl. Physiol.* 126 30–43. 10.1152/japplphysiol.00685.2018 30335577

[B43] Wakahara T. Fukutani A. Kawakami Y. Yanai T. (2013). Nonuniform muscle hypertrophy: its relation to muscle activation in training session. *Med. Sci. Sports Exerc.* 45 2158–2165. 10.1249/MSS.0b013e3182995349 23657165

[B44] Wakahara T. Miyamoto N. Sugisaki N. Murata K. Kanehisa H. Kawakami Y. (2012). Association between regional differences in muscle activation in one session of resistance exercise and in muscle hypertrophy after resistance training. *Eur. J. Appl. Physiol.* 112 1569–1576. 10.1007/s00421-011-2121-y 21858666

